# A rare case of spontaneous renal rupture caused by ANCA - associated vasculitides

**DOI:** 10.1590/S1677-5538.IBJU.2018.0119

**Published:** 2018

**Authors:** Yongquan Yu, Jiatong Li, Hongjun Hou

**Affiliations:** 1 Department of Radiology, WeiHai Central Hospital, Affiliated Hospital of Qingdao University, China; 2 Taishan Medical University, China

## CASE PRESENTATION

Anti - neutrophil cytoplasmic antibodies (ANCA) associated vasculitis (AAV) are severe multisystem diseases characterized by necrotizing inflammation of small blood vessels, few or no immune deposits, and circulating ANCA with specificity, chiefly against myeloperoxidase (MPO) or proteinase – 3 (PR – 3) (1).

**Figure 1:**
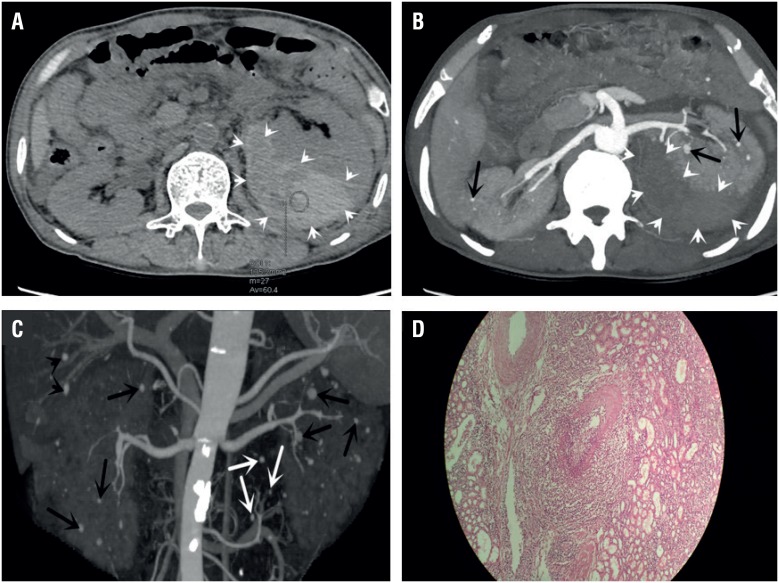
A) Non-enhanced CT shows a mass (white arrowheads) around the left kidney, which is crescent and sharply marginated with un-homogeneously high attenuation (60.4 Hounsfield); B) Axial-MIP shows a large left subcapsular perinephric hematoma (white arrowheads) and renal aneurysms (black arrows); C) 3D-removing bone MIP of the kidneys shows disseminated bilateral intrarenal microaneurysms (black arrows), multiple intrahepatic microaneurysms (black arrowheads) and scattered mesenteric arterial circulations microaneurysms (white arrows), which are like hanging nuts on twigs; D) Segmental fibrinoid necrosis of small vessels without necrotic granuloma. There are multinuclear leukocytes and monocytes infiltration in arteriole (hematoxylin and eosin, × 100).

A 61 - year - old man with hypertension for about 15 years was admitted to hospital with a 4 -hour history of left lumbago. On presentation, he was afebrile and the blood pressure was 162 / 84 mmHg. Physical examination revealed painful percussion on upper left lumbar region. Complete blood count showed leukocytosis (28.9 × 109 / L) with neutrophil (89.9%) elevation and decreased haemoglobin (60 g / L). Dynamic contrast enhanced computed tomography (CT) was performed and showed a large left - sided perinephric hematoma, multiple saccular microaneurysms were also observed in bilateral renal parenchymal, as well as in hepatic and mesenteric arterial circulations (2) (Figure-1). Then, left nephrectomy and retroperitoneal hematoma removal were performed in emergency. Histology showed segmental fibrinoid necrosis of small vessels without necrotic granuloma. Polymorphonuclear leukocytes and monocytes infiltration were found in arterioles, capillaries and venous walls with thrombus formation observed. The following laboratory test found high levels of ANCA (1: 128) and MPO (+ + +), while other specific antibodies were negative.

According to the patient's symptoms, immunological, pathological and radiologic manifestations, the disease can be identified and this patient is indeed suffering from microscopic polyangiitis (one type of specific ANCA positive vasculitis). Microscopic polyangiitis needs to be differentiated from granulomatosis with polyangiitis and eosinophilic granulomatosis with polyangiitis (other types of specific ANCA positive vasculitis), as well as from polyarteritis nodosa. Pathohistological examinations show no pulmonary nodules or granulomatous inflammation in this case, so the possibility of the granulomatous polyangiitis is excluded. On the other hand, eosinophilic granulomatosis with polyangiitis is characterized by asthma, peripheral and tissue eosinophilia, however, the symptom and the results of laboratory examination of the patient did not support it. Besides, the level of pANCA is very low in patients with polyarteritis nodosa, but microscopic polyangiitis is usually strongly positive for pANCA. This is the main point of identification of these two diseases.

To the best of our knowledge, this is a rare case causing multiple aneurysms in the renal, hepatic and mesenteric arterial circulations associated with AAV (3). Moreover, it is possible that hypertension might also has a role in contributing to this undesirable result.

### Statement

The study was approved by the ethics committee of WeiHai Central Hospital (NO. WH2017 – 66) in 8th March, 2017 and written informed consent to publish the material was obtained from the patient.
